# Determination of Flibanserin in Female Sexual Desire Enhancer Products by LC–MS/MS and Its Confirmation by LCMS-IT-TOF

**DOI:** 10.1021/acsomega.5c13004

**Published:** 2026-02-04

**Authors:** Abeer Elriş, Mazlum Akif Altun, Saniye Özcan, Serkan Levent, Nafiz Öncü Can

**Affiliations:** † Department of Analytical Chemistry, Graduate School, 52944 Anadolu University, 26470 Eskisehir, Turkiye; ‡ Department of Analytical Chemistry, Faculty of Pharmacy, Anadolu University, 26470 Eskişehir, Turkiye; § Central Analysis Laboratory (MERLAB), Faculty of Pharmacy, Anadolu University, 26470 Eskişehir, Turkiye

## Abstract

Hypoactive sexual desire disorder, a common condition among females, often goes undiagnosed due to the lack of available pharmaceutical treatments or the stigma associated with this condition. Flibanserin was initially investigated as an antidepressant but failed to demonstrate efficacy for depression; however, subsequent clinical studies revealed its potential to enhance sexual desire in some individuals, leading to its approval by the U.S. Food and Drug Administration in 2015 for the treatment of hypoactive sexual desire disorder. In this study, a novel LC–MS/MS method was developed and validated for the determination of flibanserin in commercial products marketed in Türkiye as female sexual desire enhancers. Chromatographic separation was achieved using a SunShell C18 column (100 mm × 4.6 mm, 2.6 μm), with a mobile phase consisting of 0.1% formic acid in methanol and 0.1% formic acid in water (65:35, *v*/*v*). The method exhibited a low limit of quantification (1.00 ng/mL) and a short analysis time of 5 min. The method was fully validated in accordance with the ICH Q2­(R2) guideline. Accuracy studies performed on samples in which flibanserin was not detected yielded recovery values between 90.04% and 94.60%. Interday linearity studies conducted over 3 days showed no statistically significant differences (*p* = 0.9823, ANOVA). Commercial samples collected from various sources were analyzed using both LC–MS/MS and LC-MS-IT-TOF techniques, revealing the absence or very low levels of flibanserin in the investigated products, raising concerns regarding product authenticity and potential risks to women’s health. In addition, the proposed analytical approach was evaluated using greenness, blueness, and whiteness metrics, demonstrating its suitability as a sustainable and applicable method for routine control analyses.

## Graphical abstract

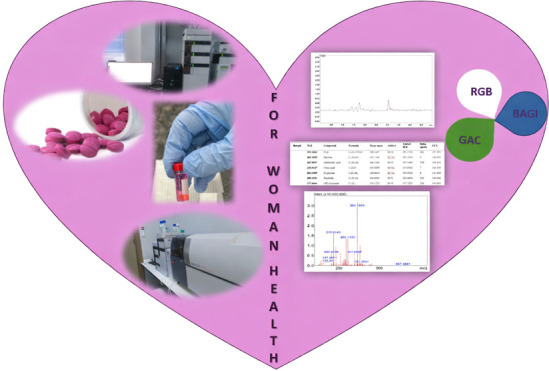

## 1 Introduction

In the world, many people experience sexual problems at some point in their lives. Women are disproportionately affected due to the lack of appropriate treatments in modern medicine, leading to most of them not receiving treatment or finding an appropriate solution. The research showed that one in every 10 women in the United States has some form of sexual dysfunction.^1^ Flibanserin (FLB), an active substance that can be used especially in hypoactive sexual desire disorder (HSDD), has become a source of hope for many women with this problem.

FLB was initially developed as an antidepressant; however, due to FLB’s inability to demonstrate an antidepressant effect in the experiments, participants indicated heightened sexual desire as a side effect, suggesting the potential application of this substance in the treatment of HSDD. So the Food and Drug Administration (FDA) approved FLB again in 2015, but this time for the treatment of HSDD instead of depression.^2^ According to the data obtained from the begonia, daisy and violet studies, 12.8% of those taking FLB experienced side effects such as drowsiness, 10.6% dizziness, 9.9% nausea and 7.1% fatigue.^3^ Due to these side effects, it is recommended that FLB be taken before going to bed. Also, taking FLB with alcohol was avoided due to the risk of fainting and hypotension.

Although flibanserin (FLB) is one of the few active substances approved for the treatment of hypoactive sexual desire disorder, the number of available studies remains limited, as its approval by the FDA is relatively recent. The majority of published studies have focused on pharmaceutical quality control or bioanalytical applications, predominantly employing LC–MS/MS^4–10^ and HPLC^4,11^ techniques. These studies have investigated a wide range of sample matrices, including natural products such as beer, grape wine, and herbal tea, as well as biological samples (e.g., plasma and brain tissue) and pharmaceutical formulations such as Veroxeserin. In contrast, a study conducted in Poland reported the presence of FLB in certain herbal products, raising concerns regarding the unauthorized use of this compound.^7^ This finding prompted questions about the potential presence of FLB in products marketed as female sexual desire enhancers in the Turkish market. Therefore, the present study aimed to determine FLB in various commercially available products obtained in Türkiye to assess the presence of FLB and to characterize their contents. Notably, it was observed that some products lacked manufacturer address information, despite regulatory requirements, and that the recommended usage instructions were concerning, particularly in light of the known side effects of FLB.

This study aimed to develop and validate a sensitive and reliable LC–MS/MS method for the determination of flibanserin (FLB) in commercial products collected from the Turkish market that are marketed as female sexual desire enhancers. The samples were additionally analyzed using an LC-MS-IT-TOF instrument to support the qualitative characterization of their contents. A further objective of this work was to investigate whether FLB is being inappropriately incorporated into over-the-counter products in a manner analogous to the illicit use of sildenafil in food products or dietary supplements. By screening products predominantly obtained through online sales channels, this study sought to assess the potential unauthorized presence of FLB and to highlight possible risks to women’s health associated with such unregulated practices. As a complementary aspect, the environmental and practical performance of the proposed analytical approach was evaluated using greenness, whiteness, and blueness assessment tools, including ComplexMoGAPI, AGREE, WAC, and BAGI.

## 2 Experimental Section

### 2.1 Reagents and Chemicals

Acetonitrile and methanol were purchased from J.T. Baker (USA) and Merck KGaA (Germany), respectively. Formic acid was purchased from Fisher Chemicals (USA) and FLB reference standard was purchased from Biosynth (USA). All solvents were LCMS grade.

### 2.2 Instrumentation

The mass spectrometric studies were succeeded in an 8040 model MS/MS instrument connected to a Nexera XR Series LC (from Shimadzu); the whole system was composed with DGU-20A3R degasser, LC-20AD gradient pump, SIL-20AC autosampler, CBM-20A communications bus module, CTO-10ASVP column oven.

The LCMS-IT-TOF series liquid chromatography–high-resolution mass spectrometry instrument (Shimadzu) was used for structural characterization; the instrument consisted of the following modules: DGU-20A3 degasser, 2× LC-20AD gradient pump, SIL-20A autosampler, CTO-10ASVP column oven, CBM-20A communication module, and ion-trap and time-of-flight (IT-TOF) mass spectrometer. LCMS Solutions 3.80 software was used for setting instrumental parameters and spectrum integration.

### 2.3 Preparation of the Mobile Phase, Standard Solutions, and Recovery Study Solutions

Details on the preparation of the mobile phase, standard solutions, and recovery study solutions are provided in the Supporting Information S1.

### 2.4 Sample Preparation

Products on the Turkish market that were considered likely to contain FLB and were marketed as sexual desire enhancers for women were randomly collected from both physical retail markets and online shopping Web sites for analysis. Five of the products were purchased from Istanbul-based local markets; however, these products were also commercially available through online sales platforms. The remaining products were obtained directly from online vendors. Although physical sampling was centered in Istanbul, all selected products were accessible to consumers throughout Türkiye via nationwide online sales platforms. All samples were in dropper liquid form. It was determined that some of these products had a manufacturer’s address, while others did not. The content information was also not available for every product. The content information written on the box by the manufacturers is given in Table S1. For legal reasons, code names were used instead of the manufacturer and product names. These samples were analyzed by both LC–MS/MS and LCMS-IT-TOF techniques. All samples were prepared in the same way without a complex procedure for both instruments. Each sample was diluted 3-fold with methanol. After that, it was vortexed for 5 min and kept in an ultrasonic bath for 30 min. It was filtered through a PTFE (22/25 mm, 0.22 μm) filter and subjected to analysis.

### 2.5 LC–MS/MS and LCMS-IT-TOF Conditions

The conditions for the mass spectrometer (MS) were optimized as follows: a drying gas flow rate of 15 L/min (nitrogen), a nebulizing gas flow rate of 3.0 L/min (nitrogen), a collision gas of argon, a CDL temperature of 250 °C, and a heat block temperature of 450 °C. Throughout all analysis, the multiple reaction monitoring mode was employed. The mass spectrometer was utilized with electrospray ionization in positive mode multiple reaction monitoring, operating within a mass range of *m*/*z* 100 to *m*/*z* 800. The mass transition ion-pair has been followed as *m*/*z* 391.15 → 161.05, *m*/*z* 391.15 → 119.05, and *m*/*z* 391.15 → 133.05. Using the standard FLB, the electrospray ionization settings were optimized. For quantitative evaluation, the peak area obtained from the summed ion chromatogram of the monitored MRM transitions was used in the calculation of FLB concentrations.

### 2.6 Chromatographic Conditions

SunShell C18 (100 mm × 4.6 mm, 2.6 μm particles) column was used as stationary phase column set at 30 ± 0.1 °C. The autosampler temperature was 15 ± 0.1 °C. Mobile phase was %0.1 formic acid in MeOH: %0.1 formic acid in water (65:35, *v*/*v*) with the flow rate of 0.5 mL/min; The injection volume was 0.3 μL with isocratic mode.

### 2.7 Method Validation

The validation of the developed method was tested as specified in the ICH Q2­(R2) guideline and was shown to meet the analytical criteria. Method validation was confirmed by detection limit, quantification limit, linearity, precision, accuracy, stability, and robustness tests. The system suitability tests (SST) were calculated, and they were acceptable according to United States Pharmacopeia (USP). The SST results are given in Table 1.

**Table 1:** Results of the System Suitable Tests

	calculated value	accepted value^b^
retention time (min) ± CI^a^	3.11 ± 0.003	-
relative standard deviation (%) of retention time (*n* = 8)	0.12	RSD ≤ 1%
precision for the area (*n* = 8)	2.24	RSD ≤ 1%
tailing factor	1.38	0.8 ≤ *T* ≤ 1.8
number of theoretical plates (*N*)	4290.33	*N* > 2000
HETP	34.99	-
USP width	0.19	≤1
injection precision for retention time (*n* = 6)	0.15	RSD ≤ 1%

a Confidence interval at 95% confidence level.

b United States Pharmacopeia.

#### 2.7.1 Determination and Quantification Limits

The limit of quantification (LOQ) is the lowest concentration that can be measured with appropriate sensitivity and accuracy under specified analytical conditions, whereas the lower limit of detection (LOD) expresses the lowest concentration of the analyte that can be detected under the same conditions. In this study, LOD and LOQ values were experimentally determined at 3:1 and 10:1 signal/noise ratios, respectively.

#### 2.7.2 Linearity

The linearity of a method is defined as the ability to provide measurement results that are directly proportional to the concentration of the analyte. For this work, the working range was between 6.0 and 240 ng/mL. The calibration was repeated three times on different days, and the variation between the results was calculated using one-way ANOVA analysis.

#### 2.7.3 Precision

Determination of precision is divided into three categories: repeatability, intermediate precision, and reproducibility. For the developed method, the analyte solution was analyzed eight times for intraday and repeatedly eight times for three different days. For the obtained data, standard deviation, relative standard deviation, mean standard error, mean, and 95% confidence level were statistically calculated.

#### 2.7.4 Accuracy

The accuracy of the analytical method can be found by measuring how much the value found as a result of the analysis deviates from the true value. It is an important parameter to show the capability of the method when it is used in real sample analyses by calculating the recovery. In this aim, FLB standard solutions were prepared at final concentrations of 96, 120, and 144 ng/mL, and the analyses were performed in triplicate. Then, the results were calculated with the mean, standard deviation (SD), relative standard deviation (% RSD), recovery, and confidence interval at a 95% confidence level.

#### 2.7.5 Stability

The stability of the method was determined in terms of the short-term, long-term, and three freeze–thaw cycles. The short-term contains analyzing the same solution after 24 and 48 h; the long-term was done by analyzing the sample after keeping it for 3 weeks at −20 °C temperature. Stability was evaluated by calculating the change in the concentration with the confidence interval at a 95% confidence level and the recovery.

#### 2.7.6 Robustness

The robustness of the method is shown by the fact that the results are not affected when the method parameters are changed in a controlled manner. This parameter was evaluated by changing some chromatographic conditions, such as the percentage of the methanol in the mobile phase (±5%), the flow rate (±5%), and the column temperature (±3%) and calculating the deviations between the results obtained at the optimized conditions and after doing the change.

### 2.8 The Assessment of Greenness and Whiteness

Different greenness assessments, such as AGREE and ComplexMoGAPI, BAGI have established the environmental sustainability of the proposed approach. The newly implemented WAC tool specifically examined the whiteness of the method.

## 3 Result and Discussion

### 3.1 Ionization Characteristics of Flibanserin

Although the LC–MS/MS system has some financial disadvantages, its ability to detect even very low concentrations makes it a suitable system for this study. Analyses were performed to determine the ionization type, and it was determined that FLB causes positive ionization. The daughter ions and collision energy observed under multiple reaction conditions using the electrospray ionization method are given in Table 2. The waiting time was determined to be 100 ms. Three daughter ions were detected, 161.05, 119.05, and 133.05 *m*/*z*. Their fragmentation paths are given in Figure 1. It is suggested that the daughter ion with the *m*/*z* value of 161.05 is fragmented by heterolytic fragmentation, the daughter ion with the *m*/*z* value of 133.05 is fragmented by heterolytic fragmentation and carbonyl release, and the ion with the *m*/*z* value of 119.05 is fragmented by McLafferty rearrangement and hydroxyl release. The product ions at *m*/*z* 161 and *m*/*z* 119 have been reported in previous studies, while the ion at *m*/*z* 133 is considered a transitional step and is formed in lower abundance than the other two.^4,6^

**Table 2:** ESI Conditions of FLB for Multiple Reaction Monitoring in Positive Mode

compound	precursor ion	product ion	Q1 pre bias (V)	CE (V)	Q3 pre bias (V)
FLB	391.15	161.05	–19.0	–31.0	–24.0
		119.05	–19.0	–53.0	–21.0
		133.05	–27.0	–54.0	–23.0

**Figure 1:**
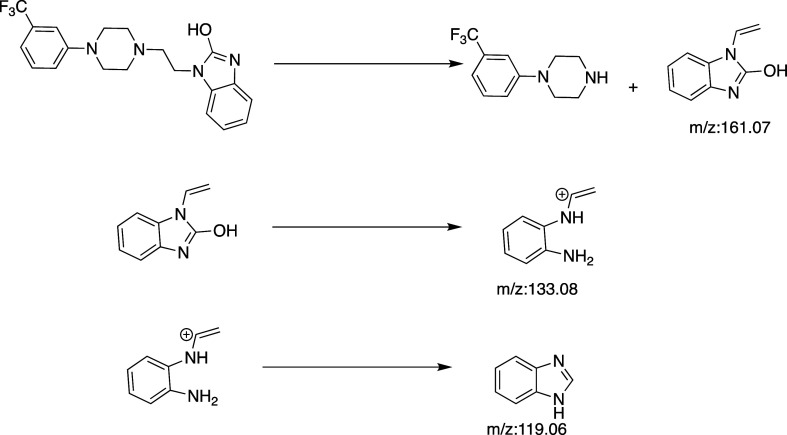
Fragmentation paths of FLB in positive electrospray ionization mode.

### 3.2 Optimization of the Chromatographic Parameters

First, studies were conducted to select the stationary phase. The C18 column was used because it has longer carbon chains, more theoretical plates, and more interaction time between the mobile phase and the packing of the column. It gave a favorable peak with a short analysis time, so it was used in the work. Second, the organic modifier of the mobile phase was selected. In the tests done with acetonitrile and methanol, FLB had a short retention time with acetonitrile. It was found that methanol produced better results. The effect of ionization agents on the analysis was also tested; the effects of formic acid and acetic acid, as well as their percentages in the mobile phase, were examined. Based on the results of the tests, a 0.1% formic acid concentration was used in both the organic and water parts of the mobile phase. After that, experiments were conducted with different ratios of organic modifier. In order to measure the effect of the column temperature, experiments were conducted at 30 °C, 35 °C, and 40 °C, and it was determined that 30 °C was the most suitable. Finally, different injection volumes (0.3, 0.5, and 1 μL) were also examined; with the high injection volume, the tailing factor of the peak was a bit high. All analyses yielded a high response and suitable SST parameters under the following chromatographic conditions: 0.1% formic acid in MeOH: 0.1% formic acid in water (65:35, *v*/*v*) as the mobile phase, C18 (100 mm × 4.6 mm, 2.6 μm particles) as a stationary phase with a column temperature at 30 ± 0.1 °C. The flow rate was set at 0.5 mL/min, and the injection volume was set at 0.3 μL. The obtained chromatograms of blank and FLB solutions are given in Figure S1.

### 3.3 Method Validation

The method was fully validated according to ICH Q2­(R2) guideline. The calculations of the validation studies were made by taking into account the responses obtained from the MS detector and the analyte concentration. First, the selectivity and specificity of the method were evaluated. The overlaid total ion chromatograms of the FLB-containing samples and blank solutions are given in the Figure S2. After detection and quantification limits were determined. Different solutions at different concentration levels were prepared and analyzed. The determination of LOD and LOQ were done according to percentage of the signals to the noise, which must equal or higher than 3/1 for LOD, and equal or higher than 10/1 for LOQ. The found LOD, and LOQ values were 0.11 ng/mL and 1.00 ng/mL, respectively. The calibration graph was created by calculating the peak area corresponding to the analyte concentration at eight levels. Where each concentration level was repeated three times and all calibration was repeated three times. The repeatability of the method was evaluated at a concentration of 120 ng/mL, which corresponds to 100% of the nominal working concentration. The repeatability was judged by the low F values and the high p values obtained from the one-way ANOVA analysis, indicating no statistically significant differences. The results of the precision and linearity studies conducted for the method are given in Table 3.

**Table 3:** Results of Linearity and Precision Studies for FLB

parameter	obtained value
working range	6.0–240.0 (ng/mL)
slope ± SD^a^ (intraday, *n* = 8)	5187.5 ± 49
intercept ± SD^a^ (intraday, *n* = 8)	7069.7 ± 6540
coefficient of determination (intraday, *n* = 8)	0.99946
slope ± SE^b^ (interday, *k* = 3, *n* = 24)	5331.4 ± 73
intercept ± SE^b^ (interday, *k* = 3, *n* = 24)	2097.1 ± 9659
coefficient of determination (interday, *k* = 3, *n* = 24)	0.99889
ANOVA	*F*(2,23) = 0.00027, *P* = 0.9997 (*P* > 0.05)
repeatability (intraday, mean, *n* = 8)	634,715
repeatability (intraday, SD^a^, *n* = 8)	2272
repeatability (intraday, RSD^c^ %, *n* = 8)	0.36
repeatability (intraday, SEM^d^, *n* = 8)	927
repeatability (intraday, CI^e^, *n* = 8)	1817
repeatability (interday, mean, *n* = 24)	633,947
repeatability (interday, SD^a^, *n* = 24)	6736
repeatability (interday, RSD^c^ %, *n* = 24)	1.06
repeatability (interday, SEM^d^, *n* = 24)	2750
repeatability (interday, CI^e^, *n* = 18)	5390
ANOVA	*F*(2,23) = 0.0179, *P* = 0.9823 (*P* > 0.05)

a Standard deviation.

b Standard error.

c Relative standard deviation.

d Standard error of the mean.

e Confidence interval at 95% confidence level.

For the accuracy study, one of the collected samples was selected, and spiked with the necessary amount FLB standard solution. These analyses were performed at three different concentrations (80%, 100%, and 120%) at nine different analyses. The recoveries ranged between 90.04% and 94.6%. The obtained data is shown in Table 4, and the obtained chromatograms are also shown in Figure 2.

**Table 4:** Data of the Recovery Studies for FLB (*n* = 3)

		precision		accuracy	
added (ng/mL)	founded ± CI^a^ (ng/mL)	SD	RSD (%)	recovery (%)	error (%)
96.0	132.2 ± 0.40	0.33	0.25	91.8	–8.2
120.0	113.5 ± 0.20	0.19	0.17	94.6	–5.4
144.0	86.4 ± 0.10	0.09	0.10	90.0	–9.9

a Confidence interval at 95% confidence level.

**Figure 2:**
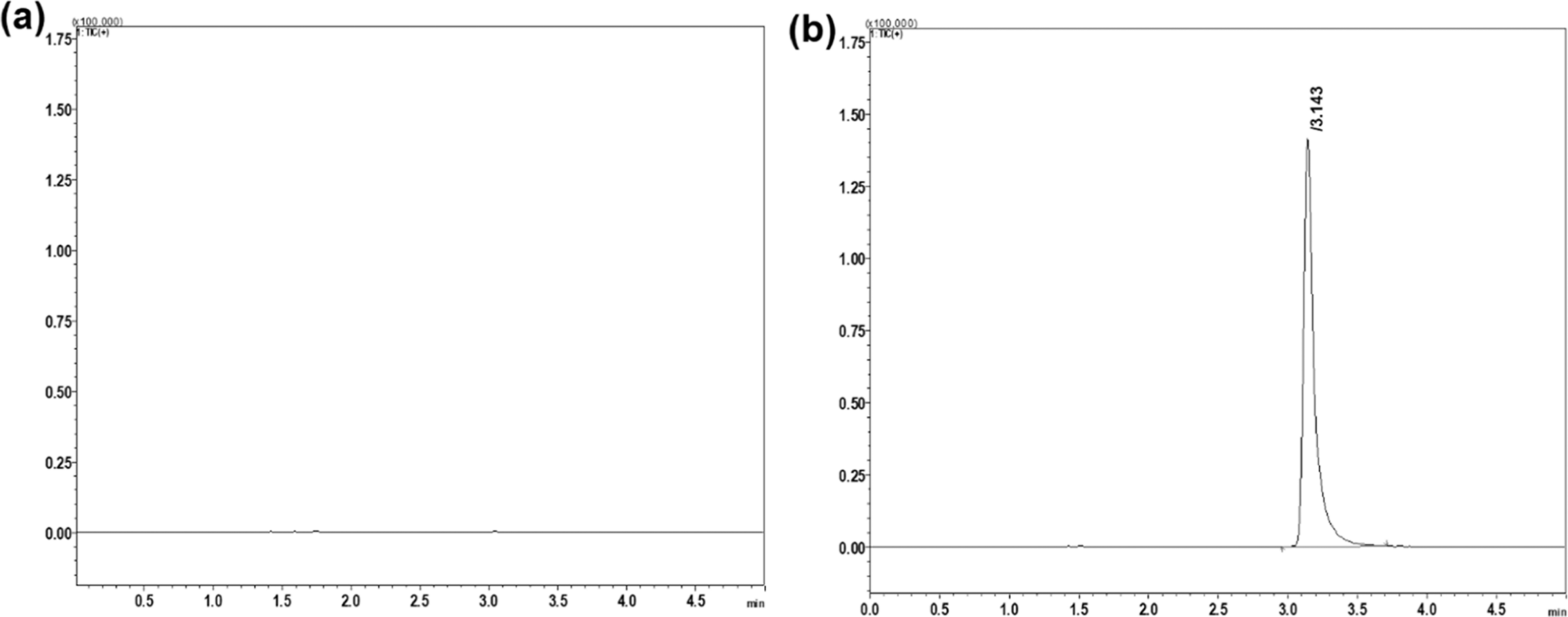
Obtained TIC of (a) the blank solution, and (b) the spiked solution of FLB standard (120 ng/mL).

The robustness tests are used to ensure that the results are not affected by small changes made in the method conditions and to determine the method limits. The retention time and peak area and differences obtained in these analysis results are calculated, and the results are given in Table S3.

The prepared FLB solutions were kept at room temperature for 24 and 48 h to examine the short-term stability and then analyzed. To evaluate long-term stability, analysis was performed after keeping at −20 °C for 3 weeks. In addition, analysis was performed for freeze–thaw stability in 3 replicates. The analyses results obtained are given in Table S3. It was evaluated at a 95% confidence interval and recovery percentages are given in the table.

### 3.4 The Greenness and Whiteness of the Developed Method

The GAPI tool assesses the environmental sustainability of a comprehensive analytical process, from sample collection to final determination, utilizing a distinct symbol comprised of five pentagrams.^12^ ComplexMoGAPI has an extra hexagonal segment that includes activities that take place before sample preparation and analysis. In this segment, it evaluates the sample preparation process in detail with parameters such as production yield, conditions, purification methods, solvent selection, instrumentation, and cumulative scores. It evaluates the overall sustainability of this method.^13^ The measurement tool provides a comprehensive score ranging from 0 to 100, providing a better qualitative and quantitative picture of environmental impact. The ComplexMoGAPI scale of the developed method was given in Figure 3a. According to the pentagram, the method’s ComplexMoGAPI score was calculated as 83. The method achieved a good score for its high accuracy, precision, and analysis speed. It is an important advantage that the samples do not require any additional purification and are prepared with simple techniques. On the other hand, it can be considered a disadvantage that they are collected from the market and brought to the laboratory for analysis. However, due to the nature of LC methods, it is not possible to transfer the method nowadays.

**Figure 3:**
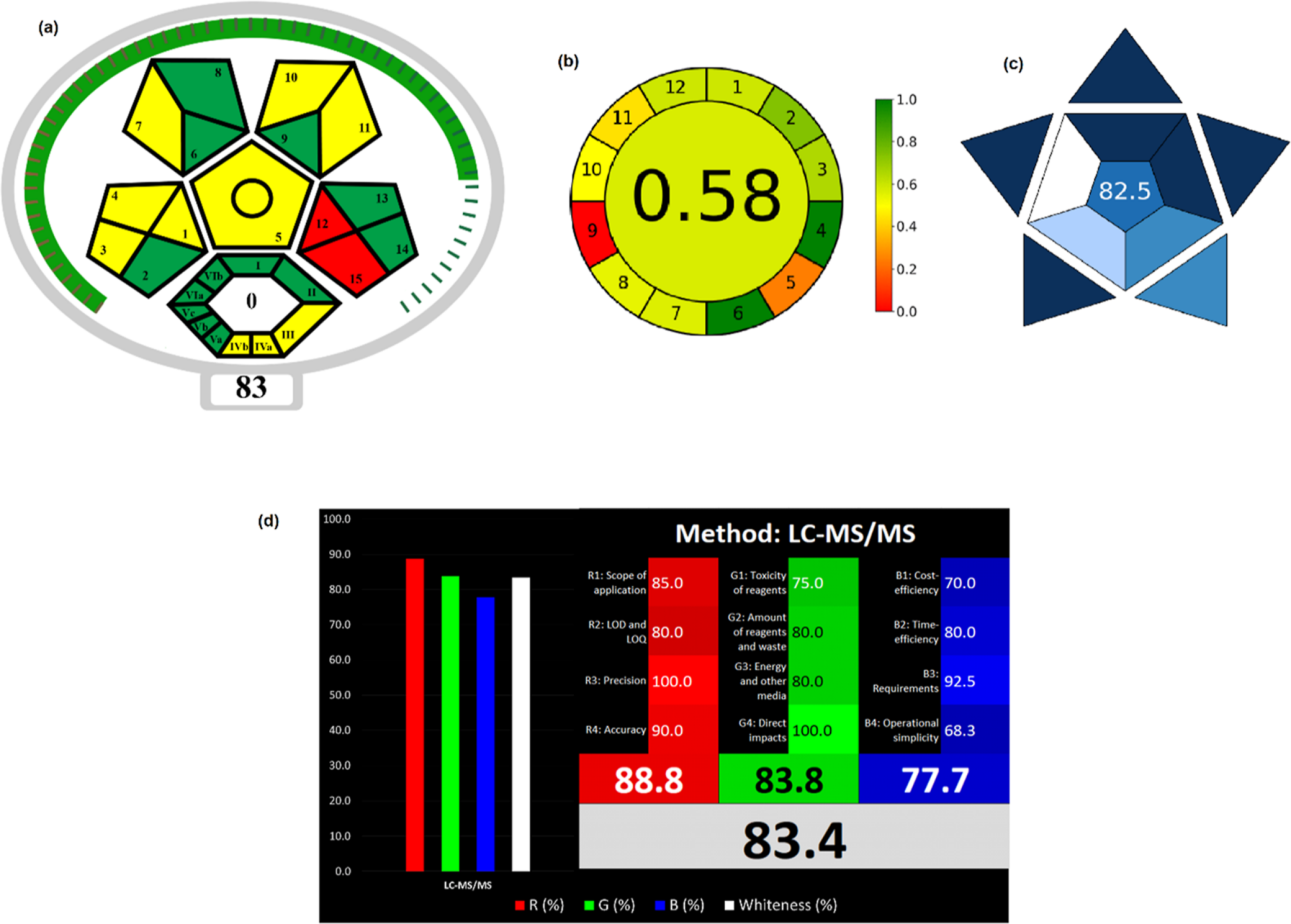
Results obtained from Complex MoGAPI (a), AGREE (b), BAGI (c) and WAC (d) tools.

The open-source AGREE program^14^ presents a circular pictogram akin to a clock, displaying 12 numbers, each representing a principle of the 12 GAC on a numerical scale ranging from 0 to 1. This scale represents a color gradient from dark green to dark red. The central figure in the AGREE pictogram, suitably colored, represents the definitive average numerical value derived from the 12 data points. The proposed LC–MS/MS method has an AGREE score of 0.58, as illustrated in Figure 3b, thus validating its environmentally friendly designation. When the quadran was examined, it waseen that the lowest score was gained from conditions numbered 9 and 5, namely energy consumption and automation and miniaturization, respectively. This is because LCMS-type devices consume a significant amount of energy, and there was no automated and integrated sample preparation procedure for the analysis system. It was unfortunately one of the general disadvantages of this type of chromatographic analysis. Meotot met other green chemistry conditions to a reasonable score.

The principal objective of the BAGI tool is to evaluate the viability of the analytical method. The pictogram illustrates the assessment as a gradient from blue to white, with dark blue signifying the optimal outcome.^15^ The BAGI graph and score (82.5) indicate that the new method demonstrates strong feasibility as shown in Figure 3c. Especially having an easy sample preparation procedure, using easily available solvents, and choosing LC–MS/MS, which was a common analysis technique, were important practical features. Moreover, a short analysis time and minimal sample preparation also seem to supported the high BAGI score.

The WAC metric tool^16^ was created to assess analytical operations from several perspectives, including efficiency (shown in red), environmental impact (green), and economic viability (blue). The notion of attaining a white result from assessing a product’s sustainability depends on the integration of the three primary colors: red, green, and blue. The approach attained an impressive score of 88.8% for red color, owing to its heightened sensitivity as seen by the limits of detection and quantification. The method achieved 83.8% for green color because to the use of a very little amount of solvent, and energy. Furthermore, it achieved a score of 77.7% for the blue criteria. The overall whiteness score is 83.4%, signifying a high level of whiteness in the procedure as given in Figure 3d. The fact that the analytical method parameters of the method were quite successful, that green chemistry recommendations were followed to the extent that the chromatographic separation efficiency allows, and that inexpensive chemical processes were preferred has provided a good whiteness score. On the other hand, the fact that the LCMS device is not an affordable analytical device that can be found in every analysis laboratory is an important limitation that reduces the whiteness score.

### 3.5 LC–MS/MS Analysis

Randomly collected ten samples were prepared as described to analyze them with the developed LC–MS/MS method. The preparation of solutions for analysis from the samples revealed significant differences in color and odor between the products claiming the same content. The obtained chromatograms of all samples are given in Figure S4. The analyses showed that although the developed method had very low detection and quantification limits, the concentration of FLB was lower than these limits in some samples and not present in others, and products that claimed to have the same content information had very different chromatograms. For example, sample 6 and sample 7 have the same content information, but the obtained mass spectra were not similar.

### 3.6 LCMS-IT-TOF Analysis

To characterization of the same samples were also analyzed in the LCMS-IT-TOF instrument. The prediction of the other contents was not easy, and some found masses were not identified. The obtained spectra are given in Figure S3, and the predicted molecules upon the masses were also given in Table S1. The prediction was done according to the obtained content lists given on the product box in the positive ionization mode using the DrugBank Web site (https://go.drugbank.com/spectra/ms/search). Because some products contain some plant extracts like licorice root extract, ginkgo biloba extract, *Panax ginseng* extract, and epimedium extract, it was especially focused on the contents of them in the study. As a result, FLB was found in very low amounts in almost all samples. Some unknown masses could possibly come from any plant extract. At the same time, these masses could belong to harmful or hazardous substances. With these results, it is thought that these products have a very low or even no effect on the women with hypoactive sexual desire disorder, and products like these should not be allowed to enter the market without checking their contents that may be harmful to human health.

### 3.7 Comparison of the Method with Data in the Literature

The analytical methods developed for FLB analysis in the literature were summarized in Table 5. The methods could be divided into two parts: quality control and bioanalytical purposes. It was obvious that all the instrumentation systems used for bioanalytical analyses presented so far were LC–MS/MS. The methods developed for quality control analyses were based on analysis with HPLC and LC–MS/MS. Apart from these, there are two other high-throughput methods that were developed for various purposes. However, these two methods have significant disadvantages, such as requiring experience and mastery for routine analyses.^17,18^ Poplawska et al. were created to analyze both FLB and tadalafil at the same time in herbal products using an LC system along with PDA, MS, and a charged aerosol detector, just like the method that was developed.^7^ They also characterized herbal preparations with LCMS-IT-TOF. Compared to their method, our method has advantages such as a working range that was approximately 100 times and LOQ was 0.5 times lower. Also, they characterized 9 compounds in herbal products with high-resolution mass spectrometry studies. Our method used high-resolution mass spectrometry to characterize all samples from 9 different market brands in detail. In addition, starting from sample collection to final analysis, the principles of less environmental harm and efficient and sustainable analytical method development were considered and evaluated with 4 different metric tools, which is another originality of the our article. Moreover, this study encourages other researchers to focus on such synthetic products and assess their impact on public health. In certain societies, these products hold a peculiar appeal: their sale remains hidden, yet their use is widespread. As a result, the table already presents a detailed comparison of all the methods developed so far.^19^

**Table 5:**
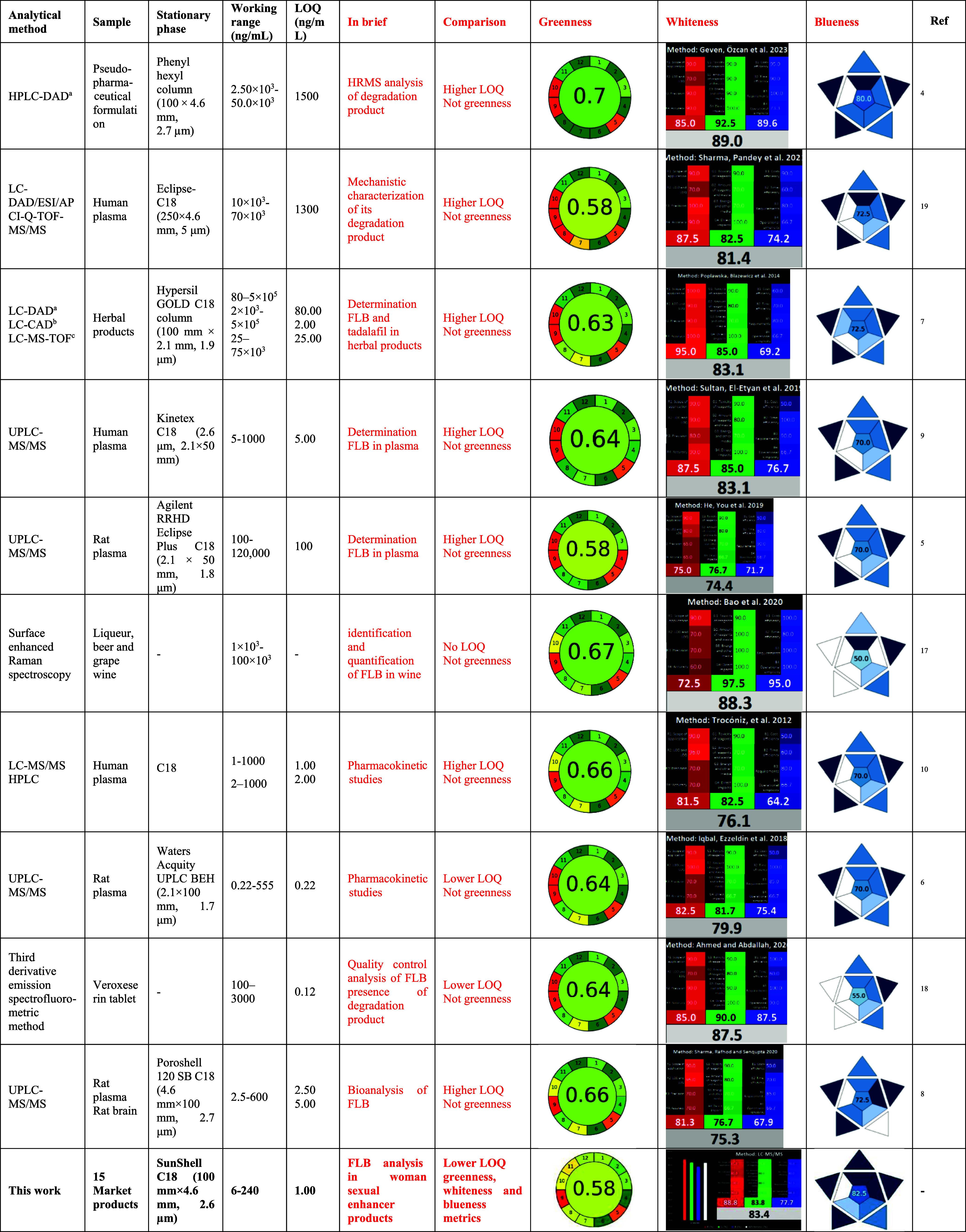
Comparison with Previous Studies

a Liquid chromatography-diode array detector.

b Charged aerosol detector.

c Mass detector.

## 4 Conclusion

Due to the lack of the drug treatment for hypoactive sexual desire disorder in women and the feeling of shame or hesitation stemming from cultural reasons. People in many countries do not think about or try to seek medical help for this issue. This situation not only complicates the diagnosis of such diseases but also exposes those affected to potential harm. Many women who cannot get medical help or access treatments like flibanserin and bremelanotide, which are available in only a few countries, often seek other options. In this case, products marketed as sexual desire enhancers for women are based on taking advantage of people’s helplessness. When marketing such products, the following is stated: “If you want to make a sweet surprise for your partner without him knowing, it will be enough to drop 7–8 drops into her drink and wait for half an hour.” Beyond the fact that such statements could lead women to take these products against their will, marketing them in this way encouraging people to use them and making it seem normal is also wrong.

FLB was licensed by the United States Food and Drug Administration as the only medication for the treatment of female sexual interest/arousal disorder of any degree.^11^ The current LC–MS/MS method is a novel, simple, and time-saving analytical method for the determination of FLB with a low quantification limit. The method was fully validated and used to determine FLB in samples that were bought as a solution to the treatment of female sexual desire enhancement. It has been shown that some of the products did not include content information. In addition, it has also been determined that the manufacturer’s address was present in some products while not others. As a result of the analysis, in some products had low concentration of FLB, which was not enough to make an effect. When the same samples were given to LCMS-IT-TOF to characterization, it was shown that the contents of the product did not match with that given in the lists on their boxes, and they included substances like caffeine, lactose, and citric acid, which have no effect on the disease., Finally, analyzing products like this in detail and identifying any substances that may threaten human health is an important point in terms of public health, especially considering that patients using such products do not have any other solution in Türkiye or in the other countries.

In the present study, a comprehensive screening of 15 finished products was conducted, making it one of the most extensive surveys of commercially available formulations reported in the literature to date. Owing to the nanogram-per-milliliter-level LOQ achieved with the developed LC–MS/MS method, the method offers not only a highly sensitive approach for the analysis of finished products but also a potential alternative for plasma and urine analyses in future bioanalytical applications. Furthermore, the method is distinguished by its strong ecological profile, demonstrated through multiple green analytical chemistry assessment tools, underscoring its suitability as an environmentally conscious analytical approach. Although the proposed method demonstrated favorable greenness, whiteness, and blueness characteristics, further enhancements in environmental performance may be achieved in future studies through the implementation of miniaturized or low-flow LC systems and the integration of automated sample preparation, which could reduce energy consumption and improve overall sustainability.

Overall, the analytical strategy presented herein provides a robust and versatile platform that can be effectively utilized across pharmaceutical quality control laboratories, food and dietary supplement testing facilities, and a wide range of academic research settings. This study therefore represents a valuable contribution to the field by combining comprehensive product surveillance with a highly sensitive, validated, and sustainability-oriented analytical methodology.

## Supplementary Material



## Data Availability

The majority of the data used to support the findings of this study are included within the article. Other data are available from the corresponding author upon request.

## Notes

Contents of Market Products’ samples specified on their boxes, The data obtained from the robustness studies of FLB, The data obtained from the stability studies of FLB, The chromatograms of blank and FLB under optimization conditions, The total ion chromatograms of all market samples obtained by LC–MS/MS (PDF)
