# Supplementary material for: Determination of Flibanserin in Female Sexual Desire Enhancer Products by LC–MS/MS and Its Confirmation by LCMS-IT-TOF
Source: ACS Omega. 2026 Feb 4;11(6):10828–36. doi: 10.1021/acsomega.5c13004 (PMC12917915; doi:10.1021/acsomega.5c13004)
Supplement: Supplementary file 1 [file ao5c13004_si_001.pdf]

SUPPLEMENTARY MATERIAL

Determination of Flibanserin in Female Sexual Desire Enhancer Products by LC-MS/MS and Its Confirmation by LCMS-IT-TOF

Author's Name: Abeer Elriş[a], Mazlum Akif Altun[b], Saniye Özcan[b,c], Serkan Levent[b,c], Nafiz Öncü Can[b,c*]

Authors Affiliations:

[a]Department of Analytical Chemistry, Graduate School, Anadolu University, 26470, Eskisehir, Turkey.

[b]Department of Analytical Chemistry, Faculty of Pharmacy, Anadolu University, 26470, Eskişehir, Türkiye.

[c]Central Analysis Laboratory (MERLAB), Faculty of Pharmacy, Anadolu University, 26470 Eskişehir, Türkiye.

E-mail addresses:

Abeer Elriş: aalreesh@anadolu.edu.tr

Mazlum Akif Altun: akifaltun@outlook.be

Saniye Özcan: saniyeozcan@anadolu.edu.tr

Serkan Levent: serkanlevent@anadolu.edu.tr

Nafiz Öncü Can: nafizoc@anadolu.edu.tr

[*]**To whom correspondence should be addressed:**

Department of Analytical Chemistry,

Faculty of Pharmacy, Anadolu University,

Yunusemre Campus, Eskisehir 26470, Turkey

Tel: +90 222 3350750 ext. 3770 Fax: +90 222 3350750

e-mail: nafizoc@anadolu.edu.tr

TABLES

Table S1. Contents of Market Products' samples specified on their boxes.

Sample	The sample contents	Ingredients found by LCMS-IT-TOF
Sample 1	Water, Sugar, L-Ascorbic acid, L-Arginine, Licorice root extract, Caffeine (8150mg/L), Vitamin B3, Potassium sorbate, Sodium benzoate	Caffeine, Vitamin B3
Sample 2	There is no content information on the box	-
Sample 3	Water, Sugar, L-Arginine, L-Ascorbic acid (Vitamin C), Caffeine, Licorice root extract, Sodium benzoate, Potassium sorbate	Caffeine
Sample 4	Water, Ascorbic acid, L-Arginine, Potassium sorbate, Sodium benzoate	Caffeine, Vitamin B3
Sample 5	Water, Sugar, L-Ascorbic acid, L-arginine, Licorice root extract, Caffeine (150mg/L), Potassium sorbate, Sodium benzoate (x)	-
Sample 6	Deionized Water, Fructose-Glucose Syrup, Licorice root extract (3.83%), Ginkgo Biloba extract (3.83%), Panax Ginseng extract (2.87%), Epimedium extract (2.87%), Acidity regulator: Citric acid, Preservative: Sodium benzoate, Sweetener: Sodium saccharin	-
Sample 7	L-Arginine, Ascorbic acid, Ginseng root extract, Sodium benzoate	-
Sample 8	Water, Sugar, L-Ascorbic acid, L-Arginine, Licorice root extract, Caffeine (150mg/L), Vitamin B3 (Nicotinamide), Potassium sorbate, Sodium benzoate	-
Sample 9	Deionized water, Fructose-Glucose Syrup, Licorice root extract (3.83%), Ginkgo Biloba extract (3.83%), Panax Ginseng extract (2.87%), Epimedium extract (2.87%), Acidity regulator: Citric acid, Preservative: Sodium benzoate, Sweetener: Sodium saccharin	-
Sample 10	There is no content information on the box	Caffeine
Sample 11	Panax Ginseng extract, Ginkgo Biloba extract, Ginger root extract, Avena Sativa fruit extract, Vaccinium vitis-idaea fruit extract, L-Argenin, L-Glycine, L-Ascorbic acid, Caffeine, Potassium sorbate, Sodium benzoate, Tartaric acid, Cinnamon Extract, Glycerin, Glucose	Caffeine
Sample 12	Panax Ginseng extract, Ginkgo Biloba extract, Ginger root extract, Oat (Avena sativa) fruit extract, Red Bearberry (Vaccinium vitis-idea) fruit extract, L-Argenine, L-Glycine (Glycine), L-Ascorbic acid (E300), Caffeine, Potassium sorbate (E202), Sodium benzoate, Tartaric acid (E334), Cinnamon Extract, Glycerin (E422), Glucose	Caffeine, L-Argenine
Sample 13	There is no content information on the box	-
Sample 14	L-arginine, Ascorbic acid (vitamin C), Sodium benzoate	Caffeine
Sample 15	Zinc gluconate, Maca root extracts, Arginine hydrochloride, Tribulus terrestris fruit extract, Guarana seed extract, Caffeine, Panax Ginseng root extract	-

Table S2. The data obtained from the robustness studies of FLB (120 ng/mL).

Changed parameter		Retention time (min)	Difference % (mean±SD[a])	Peak area	Difference % (mean±CI[b])
Flow rate (mL/min)	0.55	2.92	-8.8± 0.09	653511	-6.6 ± 0.8
	0.45	3.56	10.9 ± 0.02	749742	6.7 ± 0.7
Percentage of MeOH (%)	70.0	4.14	-16.9 ± 0.1	719378	3.2 ± 0.6
	60.0	2.67	29.0 ± 0.3	678318	-5.0 ± 0.7
Column temperature	33.0	3.24	-1.0 ± 0.3	698831	0.9 ± 0.1
	27.0	3.19	-0.3 ± 0.02	685452	-0.7 ± 0.1

[a] Standard deviation, [b]Confidence interval at 95% confidence level.

Table S3. The results of the stability studies of FLB solution (120 ng/mL).

	Founded (mean±CI[a])	Recovery (%)
**Short term stability (24 h at room temperature)**	122.3 ± 0.4	98.7
**Short term stability (48 h at room temperature)**	118± 0.7	98.3
**Long term stability (3 weeks at -20 ℃)**	118.6 ± 0.5	98.9
**The freeze-thaw cycles**	118.3 ± 0.5	98.6

[a]Confidence interval at 95% confidence level.

FIGURES

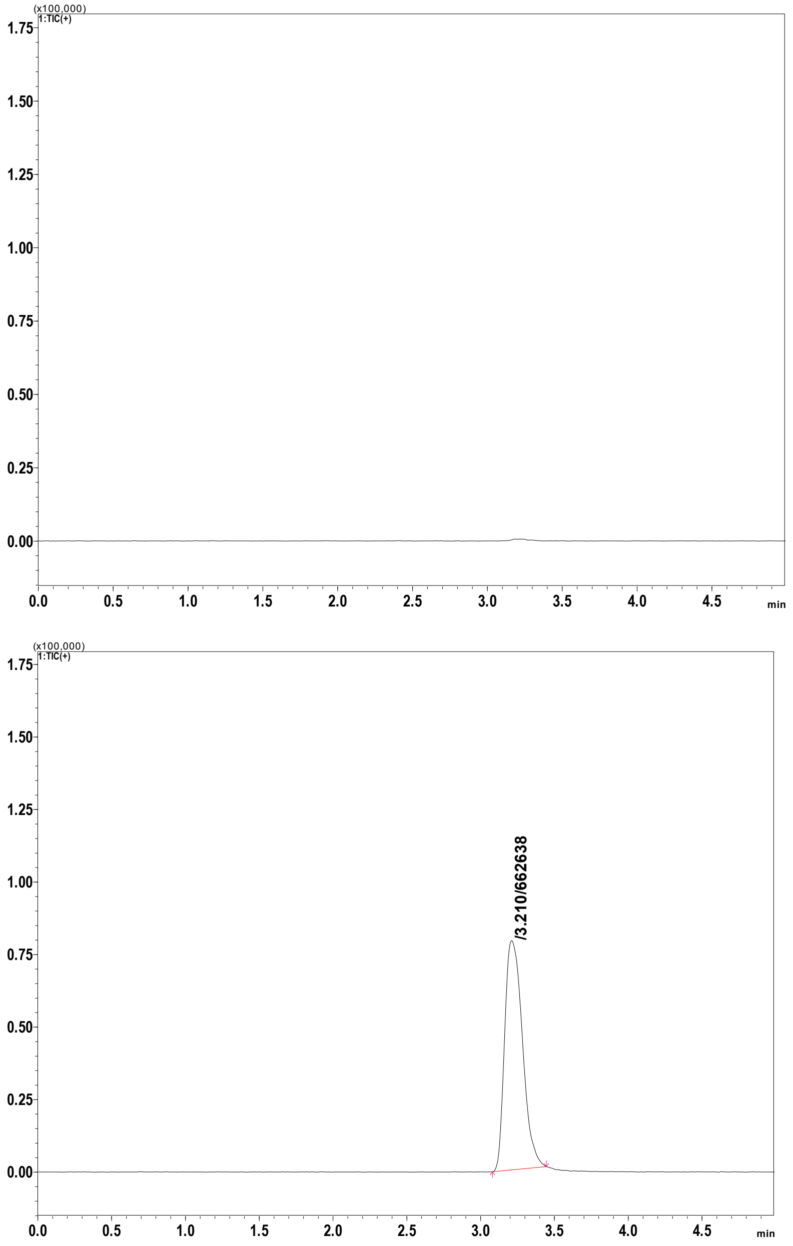

Figure S1. The chromatograms of blank (a), and FLB (120 ng/mL) (b) under the optimized conditions.

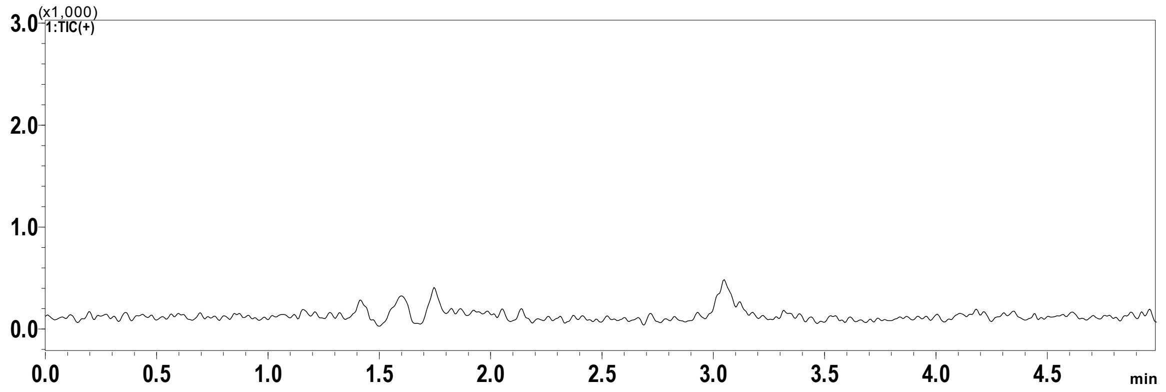

Sample 1

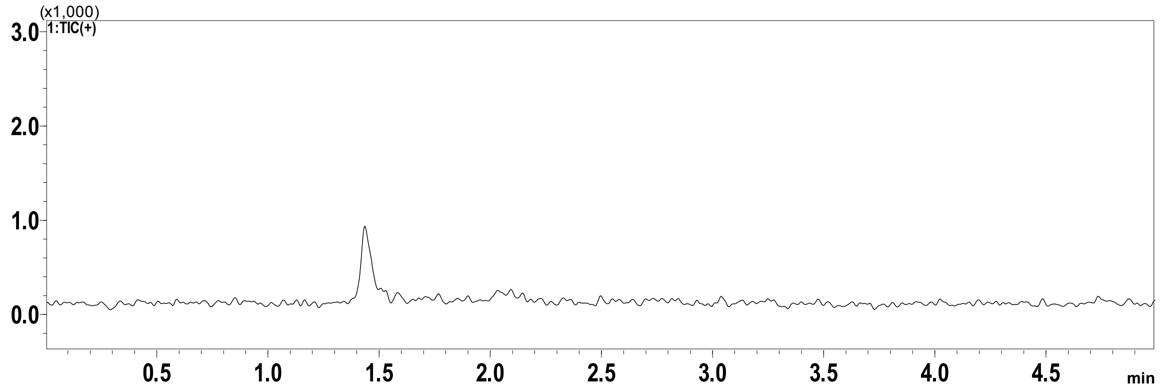

Sample 2

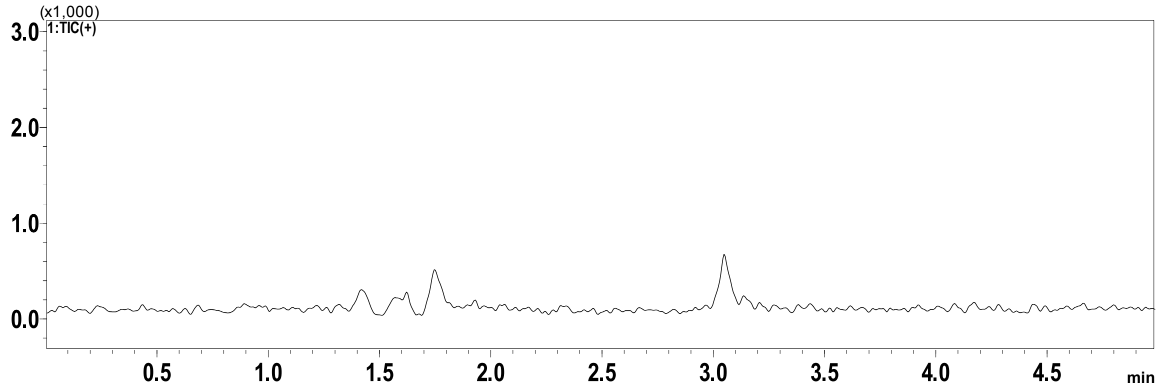

Sample 3

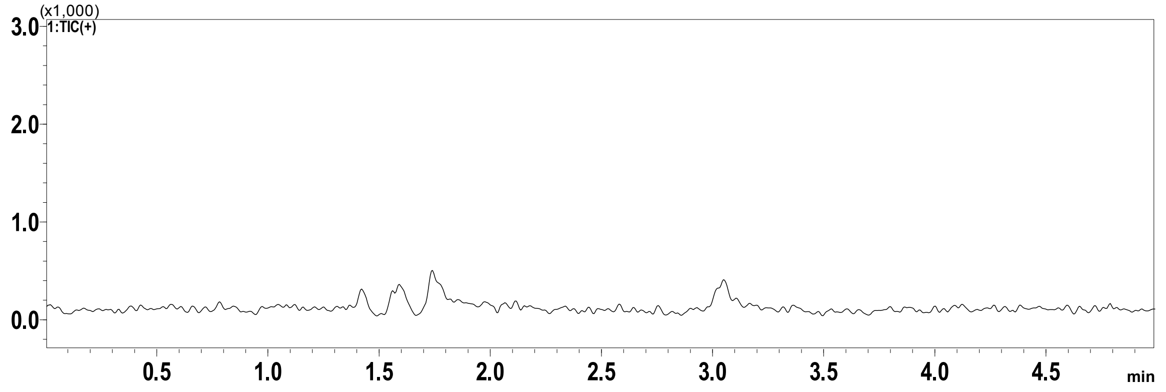

Sample 4

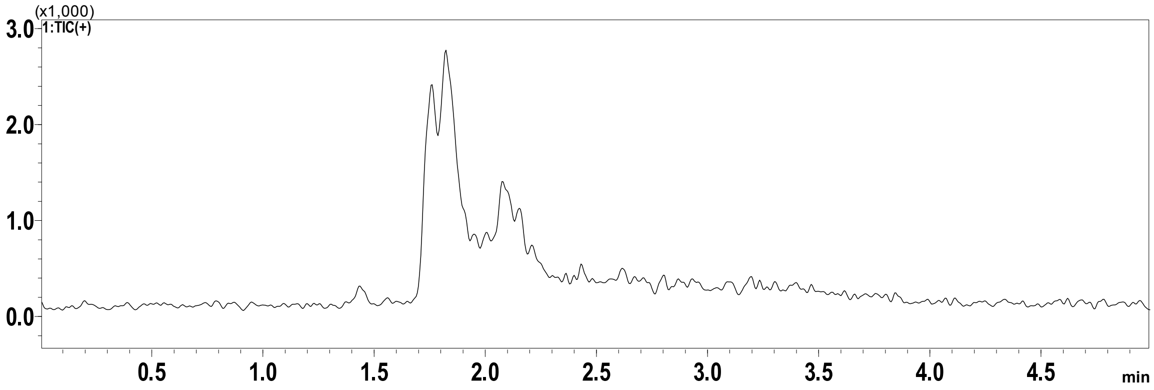

Sample 5

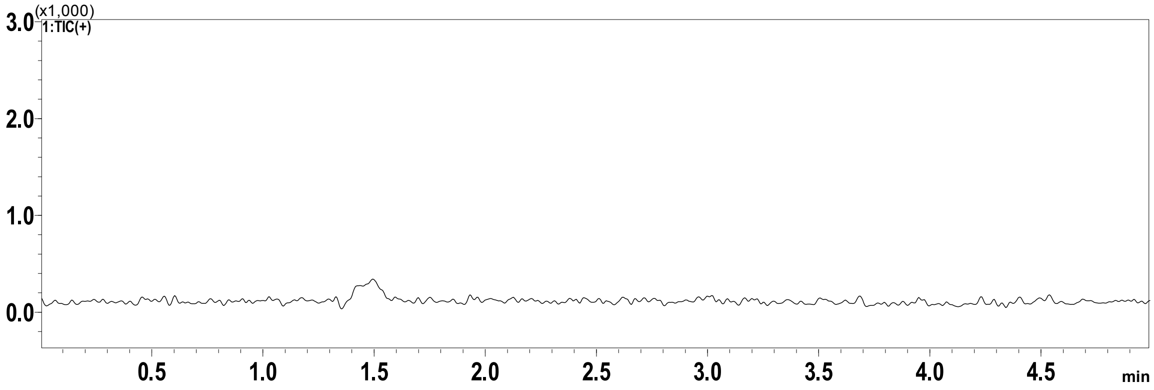

Sample 6

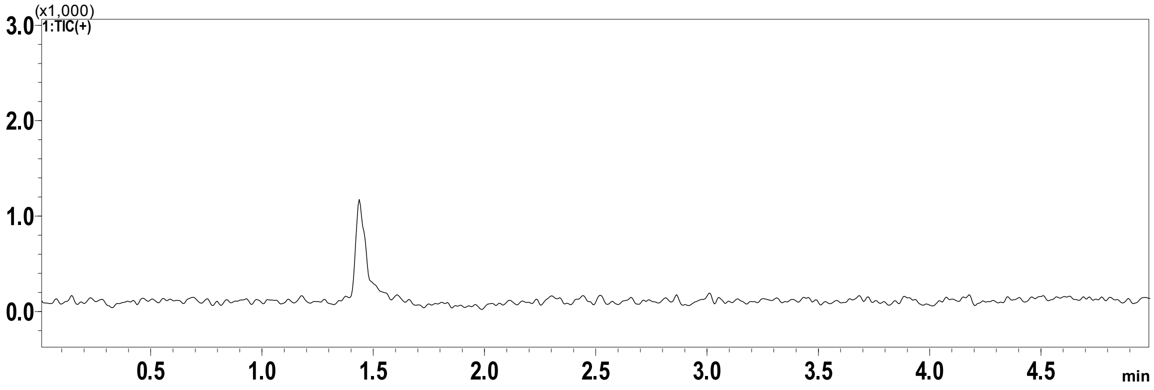

Sample 7

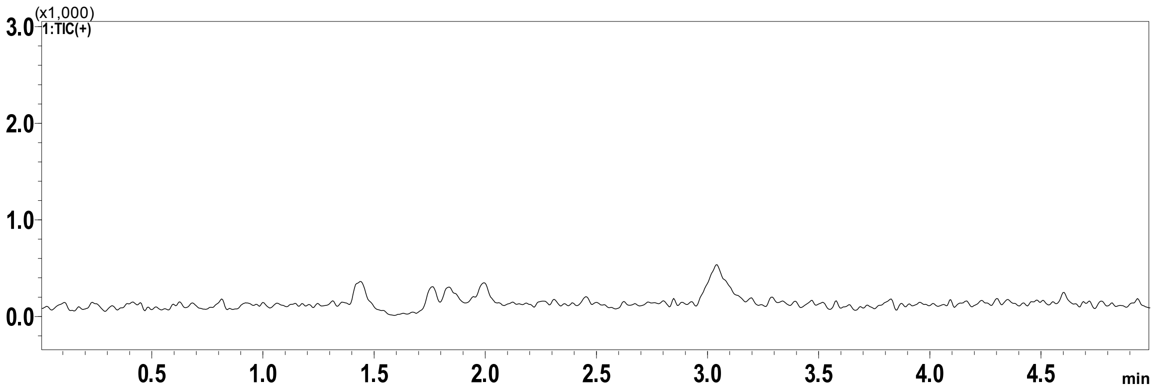

Sample 8

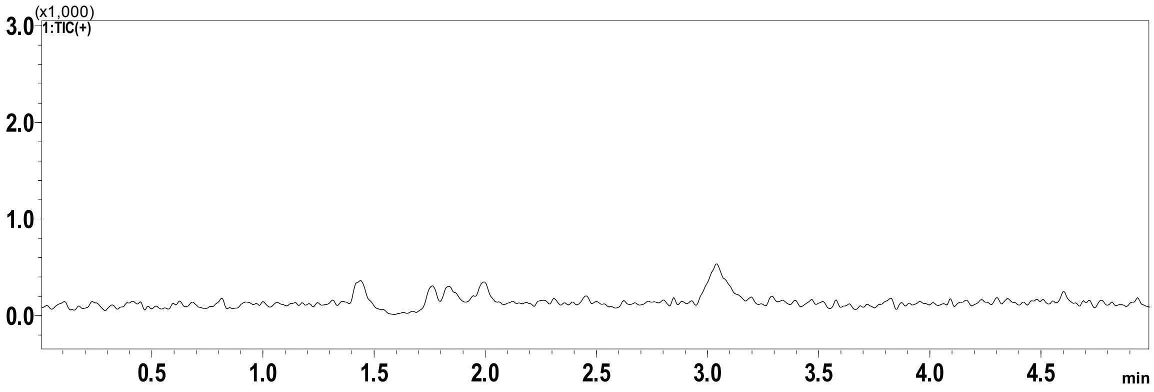

Sample 9

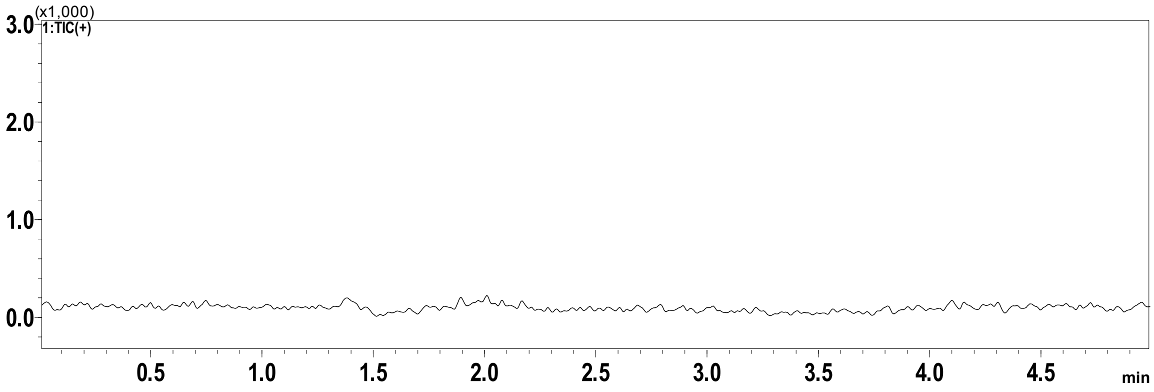

Sample 10

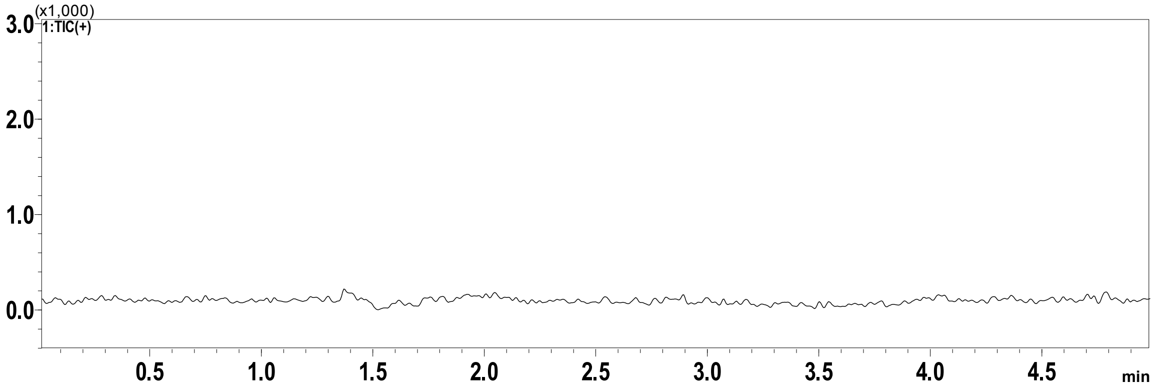

Sample 11

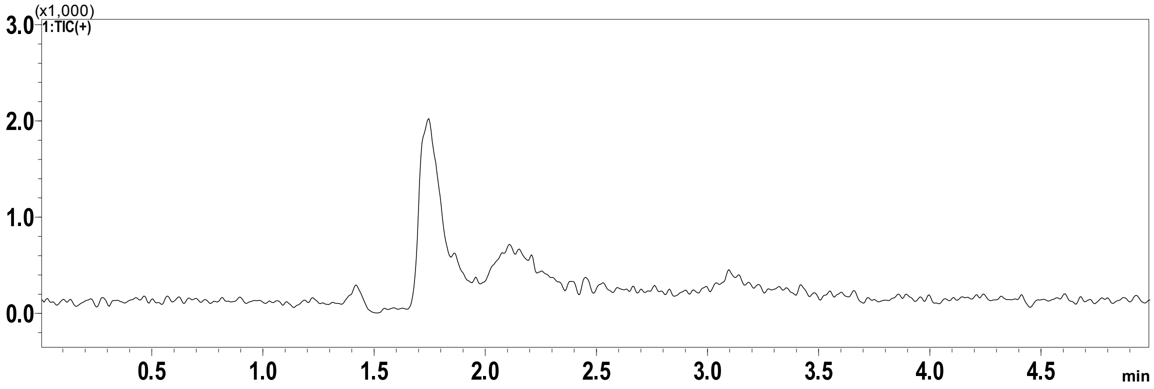

Sample 12

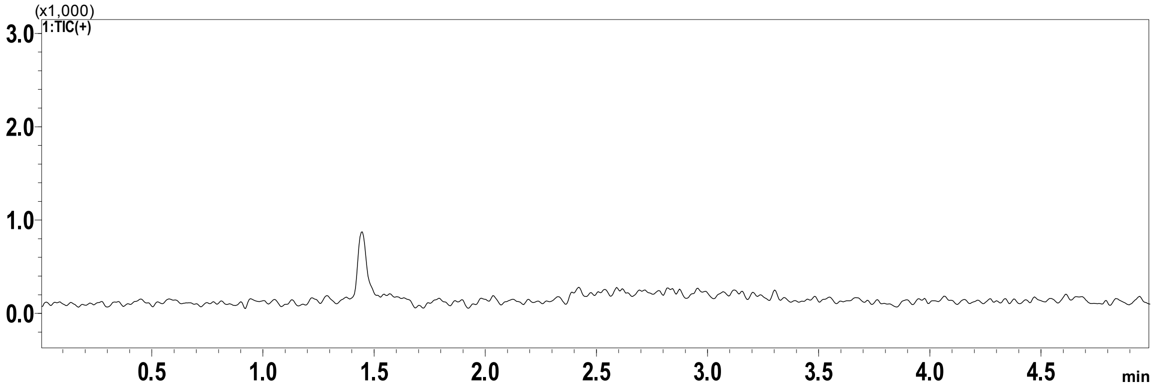

Sample 13

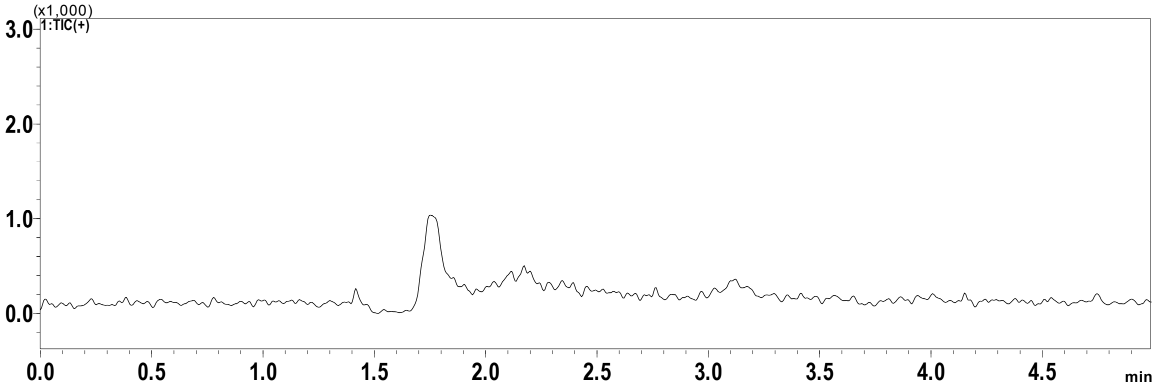

Sample 14

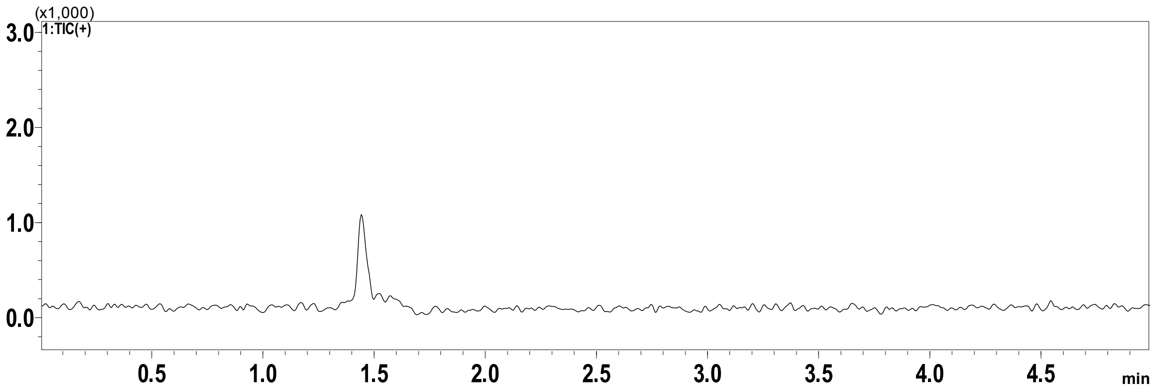

Sample 15

Figure S2. The total ion chromatograms of all market samples obtained by LC-MS/MS.

S1. Preparation of the mobile phase, standard solutions, and recovery study solutions

Recovery studies employed the standard addition method with samples in which no FLB was detected in preliminary experiments. First, 1 mL of each samples was diluted three-fold with methanol; after sonication for 15 min, the required amount of FLB standard solution was added. After that, it was kept again in an ultrasonic bath for 15 min and vortexed for 5 min. Finally, it was filtered with a PTFE (22/25 mm, 0.22 µm pore size, Isolab, Germany) filter type.

All stock solutions and working solutions of reference standards were prepared in methanol due to the solubility problem of FLB. Stock solutions of FLB were prepared by dissolving 1.5 mg in methanol to obtain a 50 µg/mL solution. Further dilutions for calibration, LOD, LOQ, and working solutions were prepared by making necessary dilutions from the stock solutions with methanol.

The mobile phase for LC-MS/MS analyses consists of methanol containing 0.1% formic acid and water containing 0.1% formic acid, both in a 65:35 (*v/v*) proportion.
